# Laser-Based Interventions for Preventing and Managing Osteoradionecrosis of the Jaw After Head and Neck Radiotherapy: A Systematic Review

**DOI:** 10.7759/cureus.98591

**Published:** 2025-12-06

**Authors:** Faisal Alzahrani, Salvatore Luca La Terra, Khyrat Y Alameer, Abdulrahman Alzahrani, Ali Alqadi, Gianluigi Caccianiga, Francesco Buoncristiani, Mario Liccardi

**Affiliations:** 1 Oral and Maxillofacial Surgery Department, Armed Forces Hospital Southern Region, Khamis Mushait, SAU; 2 Oral Surgery Department, College of Medicine and Dentistry, Ulster University, Birmingham, GBR; 3 Natural Health Science, Selinus University, London, GBR; 4 Periodontology, Private Practice, Rome, ITA; 5 Periodontology Department, College of Medicine and Dentistry, Ulster University, Birmingham, GBR; 6 Department of Translational Medicine, University of Ferrara, Ferrara, ITA; 7 Oral Surgery, Laser Dentistry, Private Dental Clinic, San Vincenzo, ITA; 8 Oral Surgery, Private Practice, Limena, ITA

**Keywords:** antimicrobial photodynamic therapy (apdt), head and neck cancer, laser therapy, low-level laser therapy (lllt), oral surgery, osteoradionecrosis (orn), photo biomodulation (pbmt), quality of life, radiotherapy complications, wound healing

## Abstract

Osteoradionecrosis (ORN) of the jaws remains a severe and functionally debilitating late complication among patients treated with radiotherapy for head and neck cancers. This systematic review evaluates the current evidence on laser-based interventions, including photobiomodulation therapy (PBMT), antimicrobial photodynamic therapy (aPDT), and surgical laser applications for the prevention and management of ORN. Ten studies met the inclusion criteria, consisting of one randomised controlled trial, one retrospective cohort study, one case series, and seven case reports. Across the available evidence, laser-based therapies were generally associated with favourable outcomes, including improved mucosal healing, reduced pain, enhanced tissue repair, and, in some cases, successful prosthetic rehabilitation and reduced recurrence. Despite these promising findings, the current evidence remains early and uneven, constrained by small cohorts, wide variation in laser parameters, limited comparative data, inconsistent staging practices, and a lack of long-term follow-up to confirm the durability of response. Laser therapy represents a potentially transformative adjunct in ORN management, but robust, well-designed clinical trials are essential to establish its true therapeutic value, optimise treatment protocols, and guide future clinical practice.

## Introduction and background

Osteoradionecrosis (ORN) of the jaws is still considered one of the most fearful late consequences of radiation in patients undergoing treatment for head and neck cancers. The presence of exposed and necrotic bone in a previously irradiated area, lasting for more than three months without any signs of tumour recurrence, leads to the clinical scenario of ORN [1]. In line with the evidence, its reported incidence varies significantly, from 2% to 22%, influenced by radiation dosage, tumour site, concurrent therapies, and oral health condition before radiotherapy [2,3]. The quality of life for patients diagnosed with ORN is dramatically compromised due to chronic discomfort, trismus, dysphagia, oro-cutaneous fistulas, and aesthetic deformities, and the treatment remains very challenging [4].

The pathophysiology of ORN has been a debated matter for decades, and in accordance with Marx’s classical theory, radiation-induced hypovascularity, hypocellularity, and hypoxia in irradiated tissues played an essential role in the onset of necrosis [5]. Current investigations emphasise the relevance of radiation-induced fibroproliferative processes, leading to increased fibrosis, chronic inflammation, and impaired tissue healing [3]. These changes determine a more difficult bone healing after an injury or infection and explain why surgical procedures like tooth extraction commonly lead to ORN in susceptible patients [1].

Conservative approaches such as antibiotics, antiseptic rinses, analgesics, hyperbaric oxygen therapy (HBOT), and surgical removal of necrotic bone all form part of traditional treatment strategies. Although HBOT has been extensively applied, its efficacy and reproducibility remain questionable [2]. More invasive surgical procedures, including segmental excision and free flap reconstruction, are mostly reserved for advanced cases; however, they are linked to considerable morbidity and extended recovery time [6].

From the perspective of minimally invasive, more efficacious alternatives, laser-based treatments have gained significant interest. Photobiomodulation therapy (PBMT), also known as low-level laser therapy (LLLT), has shown promising benefits for irradiated tissues by improving microcirculation, increasing the activity of fibroblasts and osteoblasts, reducing inflammation, and accelerating wound healing [7,8]. Clinical investigations report reduced occurrences of ORN following tooth extractions and enhanced mucosal healing and pain management in individuals with pre-existing lesions treated with PBMT [9,10].

PBMT acts through complex photochemical and photobiological pathways that remain only partially elucidated. Absorption of red or near-infrared photons by mitochondrial chromophores, notably cytochrome c oxidase, enhances oxidative metabolism and modulates secondary messengers, such as reactive oxygen and nitrogen species. These effects promote angiogenesis, fibroblast proliferation, and collagen deposition, theoretically counteracting radiation-induced hypoxia and fibrosis [11,12]. However, while in vitro and preclinical models demonstrate consistent upregulation of pro-healing cytokines and growth factors, clinical translation remains heterogeneous due to variability in wavelength, fluence, and dosimetry parameters. Similarly, antimicrobial photodynamic therapy (aPDT) employs a photosensitiser-light-oxygen triad to generate cytotoxic singlet oxygen, achieving selective microbial reduction within necrotic or irradiated fields [13]. Although mechanistically distinct, both modalities converge in mitigating infection and improving tissue perfusion, yet robust clinical standardisation and dose-response evidence remain lacking. 

Further studies have examined the alternative use of lasers for conventional debridement or sequestrectomy, such as diode, Er:YAG, and CO₂ lasers. Compared to mechanical devices, lasers exhibit various advantages, including enhanced bone removal, minimal/absent bleeding, antibacterial properties, and reduced thermal damage [14,15]. Clinical case studies demonstrate that laser-assisted surgery for advanced ORN results in accelerated healing and a reduced recurrence rate compared to conventional scalpel surgery [16,17].

Recently, an international guideline jointly published by the International Society of Oral Oncology (ISOO), the Multinational Association of Supportive Care in Cancer (MASCC), and the American Society of Clinical Oncology (ASCO) systematically reviewed the prevention and management of ORN in head and neck cancer patients treated with radiotherapy. Despite screening over 1,500 records and including 80 studies, the panel concluded that no recommendation could be made regarding the use of PBMT for the prevention of ORN, due to the limited and heterogeneous evidence [18].

Several systematic reviews have previously examined the management or prevention of ORN of the jaws. Camolesi et al. [19] systematically compared pharmacologic and surgical modalities but included only minimal studies of laser-based approaches. Sombutsirinun et al. [20] specifically reviewed laser therapy for ORN prevention but identified only four small studies and lacked analysis of therapeutic use in the established lesions. Quah et al. [21] conducted a meta-analysis of adjunctive modalities during dental extraction, including HBO, Pentoxifylline and Tocopherol (PENTO), antibiotics, and platelet concentrates. Yet, their scope was confined to preventive interventions. In addition, position papers such as those by Robijns et al. [22] have outlined PBMT parameters for radiation-related toxicities in general, but without ORN-specific outcomes. Consequently, no prior systematic review has comprehensively synthesised both preventive and therapeutic laser-based interventions (PBMT, aPDT, and surgical lasers) for ORN following head-and-neck radiotherapy. The present review addresses this gap by collating contemporary clinical evidence (2010-2025) using a PRISMA 2020-compliant methodology and structured risk-of-bias assessment.

## Review

Methods 

Aim

This study aimed to investigate the role of laser therapy in the prevention and management of ORN of the jaws in patients who have undergone head and neck radiotherapy.

Objectives

The objectives of this review are to systematically evaluate the current clinical evidence on the use of laser-based therapies for the treatment and prevention of ORN, assess their clinical effectiveness in promoting mucosal healing, reducing pain, enhancing bone regeneration, and preventing recurrence, explore their safety profile, and, where possible, compare laser-based interventions with conventional or non-laser alternatives.

Research Question

Among patients who have undergone head and neck radiotherapy, do laser-based interventions improve clinical outcomes (e.g., reduction in incidence, severity, or symptom burden of ORN) compared with conventional management approaches? The complete PICO framework (Population/Patient/Problem, Intervention, Comparison, and Outcome) for the research question is presented in Appendix A.

Eligibility Criteria

Studies were considered eligible if they reported on patients of any age or gender with a history of head and neck radiotherapy who were either at risk of developing ORN or already diagnosed with the condition. Eligible designs included randomised controlled trials (RCTs), non-randomised controlled trials, cohort studies (both prospective and retrospective), case-control studies, case series, and case reports. Interventions of interest comprised any laser-based treatment, including but not limited to low-LLT, PBMT, surgical lasers Er:YAG and diode surgical lasers, CO₂ lasers, and aPDT.

Comparators included standard care, placebo or sham treatments, and non-laser modalities such as pharmacological therapy or HBOT. Outcomes of interest were time to healing, pain reduction, mucosal coverage or bone regeneration, recurrence or progression of ORN, and adverse events. Exclusion criteria were in vitro and animal studies, narrative reviews or editorials, and studies that lacked extractable primary outcome data. Studies were also excluded if they involved patients without a history of head and neck radiotherapy, focused exclusively on prevention without ORN-related endpoints, or assessed only non-laser therapies. Duplicate publications were excluded, with only the most complete or recent version retained. Abstracts, conference posters, and protocols without available full-text data were also excluded. Table 1 summarises the inclusion and exclusion criteria.

**Table 1:** Summary of the inclusion and exclusion criteria. ORN: osteoradionecrosis, RCT: randomized controlled trial, LLLT: low-level laser therapy, PBMT: photobiomodulation therapy, Er:YAG: erbium:yttrium-aluminium-garnet, aPDT: antimicrobial photodynamic therapy, HBO: hyperbaric oxygen

Category	Inclusion criteria	Exclusion criteria
Population	Patients of any age or gender with a history of head and neck radiotherapy, either at risk of or diagnosed with ORN	Patients without a history of head and neck radiotherapy
Study design	RCTs, non-randomised controlled studies, prospective/retrospective cohort studies, case-control studies, case series, case reports	In vitro or animal studies, narrative reviews, editorials, and studies without primary outcome data
Interventions	Any laser-based therapy, including LLLT, PBMT, surgical lasers (Er:YAG, diode, CO₂), and aPDT	Studies assessing only non-laser therapies
Comparators	Standard care, placebo/sham treatments, or non-laser modalities (e.g., pharmacological therapy, HBO)	No comparator (ineligible only if combined with other exclusion criteria)
Outcomes	Healing time, pain reduction, mucosal coverage, bone regeneration, recurrence or progression, adverse events	Studies with no extractable outcome data or focused solely on prevention without ORN-specific outcomes
Publication type	Full-text, peer-reviewed studies published in English	Abstracts, conference posters, protocols without full text, and duplicate reports (less complete versions excluded)

Sources and Search Strategy

This review followed the Preferred Reporting Items for Systematic Reviews and Meta-Analyses (PRISMA) guidelines. The protocol was registered prospectively with the International Prospective Register of Systematic Reviews (PROSPERO) under registration number CRD420251091143.

A comprehensive literature search was conducted in July 2025 across PubMed, Scopus, and Web of Science. The search method integrated Medical Subject Headings (MeSH) with free-text terms such as “osteoradionecrosis,” “jaw necrosis,” “ORN,” “laser therapy,” “low-level laser therapy,” “photobiomodulation,” “photodynamic therapy,” and “CO₂ laser,” utilising Boolean operators with (AND, OR). Filters were applied to restrict findings to human research published from January 2010 to March 2025. Furthermore, the reference lists of the included studies were carefully inspected to locate additional acceptable publications. The search string can be found in Appendix B.

Data Management and Study Selection

All search results were transferred into Rayyan for deduplication and administration. Two reviewers (FA and SLL) independently screened titles and abstracts, resulting in potentially eligible studies that underwent a comprehensive full-text review. Concerns over inclusion were resolved by discussion, and if required, conciliation by a third reviewer.

Data Extraction

Data were extracted independently by two reviewers (FA and SLL) using a standardised extraction form. Extracted information included study characteristics, patient demographics, details of the intervention (laser type, wavelength, energy settings, frequency, and adjuncts), comparator or control treatments, follow-up duration, and reported clinical outcomes. The primary outcomes of interest were as follows: time to healing (mucosal or bone coverage), pain reduction (e.g., using visual analogue scales), mucosal coverage or bone regeneration (assessed clinically or radiographically), recurrence or progression of ORN (e.g., exposure extension or worsening), and adverse events (e.g., infection or complications). Where multiple outcomes or timepoints were reported, those deemed most clinically relevant or designated as primary by the original authors were prioritised. In cases of missing or unclear data (e.g., intervention settings or follow-up duration), efforts were made to contact study authors. Where this was not feasible, missing fields were marked as ‘NR’ (not reported), and no assumptions were made.

Synthesis Methods

Due to the heterogeneity in study designs, laser protocols, outcome measures, and follow-up durations, a meta-analysis was not feasible. Therefore, a narrative synthesis approach was employed. Studies were synthesised by study design. Outcomes were descriptively summarised, and tables were used to present key characteristics, results, and risk of bias. This approach allowed for a structured comparison across diverse clinical protocols.

Risk-of-Bias Assessment

Two reviewers independently assessed the quality of included studies. Any discrepancies in scoring or interpretation were resolved through discussion, with input from a third reviewer when necessary, and the assessment was conducted in accordance with the study design. RCTs were appraised using the Cochrane Risk of Bias 2 (RoB 2) tool, while retrospective/cohort studies, case series, and case reports were evaluated using the Joanna Briggs Institute (JBI) Critical Appraisal Tool [23,24]. 

Results 

Study Selection

A total of 148 records were identified through database and register searches, including PubMed (n = 6), Web of Science (n = 36), Scopus (n = 22), Google Scholar (n = 79), International Clinical Trials Registry Platform (ICTRP, n = 4), and ClinicalTrials.gov (CTR, n = 1). An additional study was identified manually through the reference list of an included article.

**Figure 1:**
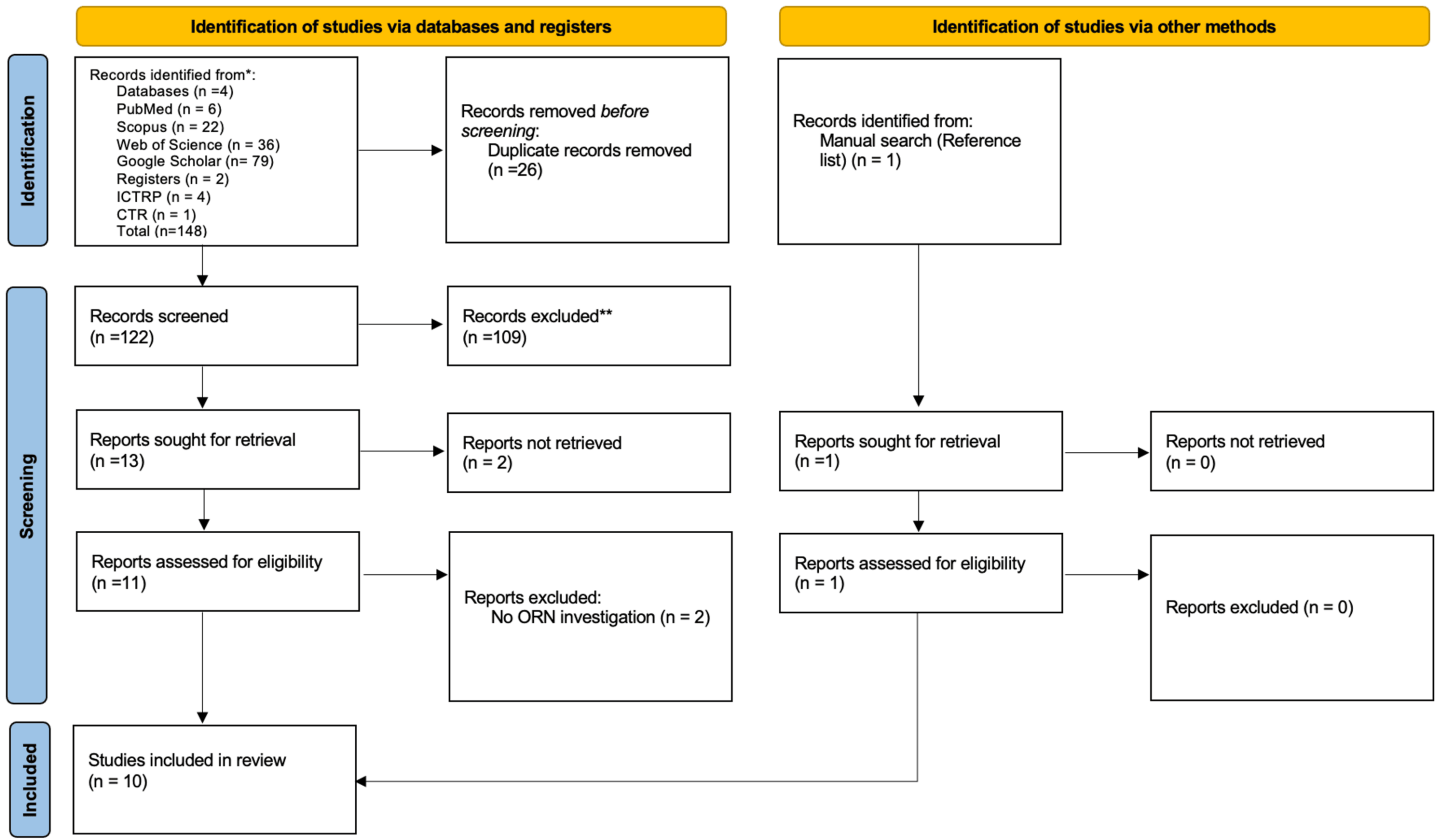
Preferred Reporting Items for Systematic Reviews and Meta-Analyses (PRISMA) 2020 flow diagram showing the selection process of studies for inclusion in this review. A total of 10 studies were finally included. Adapted from Page et al. [25]

After removing 26 duplicate records, 122 records remained for screening. Of these, 109 were excluded after title and abstract review due to various reasons such as wrong study design, unrelated outcomes, or ineligible population. 13 reports were sought for full-text retrieval. Still, two could not be retrieved due to missing or inaccessible full-texts, leaving 11 full-text articles assessed for eligibility. Of these, two were excluded for not directly addressing ORN, resulting in nine studies being included. One additional eligible study identified through manual reference search brought the total to ten included studies in this review (Figure 1).

The reasons for study exclusion are detailed in Appendix C.

Study Characteristics

A total of 10 studies were included in this review: one RCT, one retrospective study, one case series, and seven case reports, six conducted in Brazil and one in Italy, published between 2017 and 2024. Across studies, patient populations were consistently post-radiotherapy head and neck cancer patients, with radiotherapy doses ranging from 60 to 74 Gy. The mean age ranged from 53 to 69 years, with most studies reporting a predominance of male patients. Most studies had mandibular lesions and late-stage ORN, reflecting the clinical challenge of managing advanced irradiated tissue damage (Table 2).

**Table 2:** Clinical characteristics and outcomes of included studies evaluating laser-based treatments for ORN. aPDT: antimicrobial photodynamic therapy, H&N: head and neck, HPL: high-power laser, J/cm²: Joules per square centimetre (energy density), J/point: Joules delivered per irradiation point, LLLT: low-level laser therapy, MB: methylene blue, N/A: not applicable, NNT: number needed to treat, ORN: osteoradionecrosis, PBMT: photobiomodulation therapy, QoL: quality of life, RCT: randomised controlled trial, RT: radiotherapy, SCC: squamous cell carcinoma, VNS: visual numerical scale

Author	Study design	Country	Sample size (n)	Population	Site	ORN stage / criteria	Intervention (laser type, parameters, sessions)	Comparator / control	Outcomes	Follow-up	Main findings	
da Silva et al. [10]	RCT (pilot, double-blind)	Brazil	22	Post-radiotherapy H&N cancer (mean age 57; 64% male)	Mandible 13 (59.1) Maxilla 4 (18.1) Mandible and maxilla 5 (22.8)	Marx Stage I–II (68%), Stage III–IV (32%)	Diode laser 808 nm, 40 mW, 100 J/cm², 2.8 J/point, 4 sessions (weekly); adjunct: antibiotics, analgesics	Sham-PBMT (inactive 660 nm)	Healing (mucosal coverage), pain (VNS), analgesic use, infection	1 month	Healing 94.7% vs. 0% (p < 0.001); pain and analgesic use significantly reduced	
Ferigatto et al. [26]	Retrospective study	Brazil	51	Post-radiotherapy H&N cancer (median age 58; 83% male)	Posterior mandible, accounting for 69.81% of cases, followed by the anterior mandible (20.75%) and the posterior maxilla (10%).	N/A (clinical diagnosis of ORN)	Diode laser 660 nm, 100 mW, 2 J/point + aPDT (0.01% methylene blue); debridement, chlorhexidine irrigation/gel	Standard care (curettage, irrigation, antibiotics if infection)	Healing (epithelialization), pain, infection	10–115 months	aPDT protocol: 75% success (median 10 months) vs. standard care 18% (115 months); hygiene protective factor (p < 0.0001)	
Ribeiro et al. [14]	Case series	Brazil	20	Post-radiotherapy H&N cancer (mean age 59; 90% male)	Maxilla: 6 lesions (30%) Anterior maxilla: 1 lesion (5%) ​ Posterior maxilla: 5 lesions (25%) ​ Mandible: 14 lesions (70%) Anterior mandible: 7 lesions (35%) ​ Posterior mandible: 7 lesions (35%)	He et al. [27] classification (14 mandible, 6 maxilla) Stage I: 8 lesions (40%) ​ Stage II: 7 lesions (35%) ​ Stage III: 5 lesions (25%)	LLLT (660 & 808 nm, 100 mW; 10–40 s/point) + aPDT (0.01% MB, 4 min pre-irradiation, 660 nm, 40 s); adjunct: chlorhexidine mouthwash	None	Healing (mucosal coverage), pain, infection, fistula remission	2 years	100% clinical improvement; 80% total mucosal coverage; Stage I healed avg. 17 wks, Stage II 8 wks, Stage III 19 wks	
Porcaro et al. [28]	Case report	Italy	1	69 y.o. male; previous squamous cell carcinoma of the left soft palate (treated 2 years earlier); received 2D head and neck radiotherapy (90 Gy over 2 months)	Posterior Maxilla	N/A (clinical diagnosis of ORN)	Er:YAG laser, 5 W CW, 250 mJ, 20 Hz; single surgical session. Adjunct: amoxicillin–clavulanic acid 875/125 mg every 12 h, from 48 h pre-op until 5 days post-op	None	Healing, pain, infection, bone exposure	12 months	Complete resolution of ORN at 12 months; no reported infection or recurrence	
Magalhães et al. [9]	Case report	Brazil	1	58 y.o. male, post-RT SCC, Stage III ORN	Anterior mandibular	Marx Stage III (bone exposure, fistula, purulence)	aPDT (MB 0.01%, 660 nm, 100 mW, 90s/point) + LLLT (660 nm, 100 mW, 10s/point); weekly after surgery	None	Healing, recurrence prevention	1 year	Successful healing, no recurrence/new lesion	
Pedroni et al. [29]	Case report	Brazil	1	57 y.o. male, smoker, diabetic, laryngeal cancer, ORN + xerostomia	Posterior mandible	N/A Clinical diagnosis (no staging system)	PBMT (660 nm, 100 mW, 10s, 1 J/point; 808 nm for xerostomia) + aPDT (MB 0.01%, 660 nm, 20s, 2 J/point); frequent sessions	None	Healing, pain, xerostomia, mucosal repair, bone regeneration	6–12 months	Controlled ORN, reduced xerostomia, imaging showed bone formation; improved QoL	
Tateno et al. [30]	Case report	Brazil	1	62 y.o. male, post-RT, tobacco/alcohol history, high risk ORN after dental extraction	Posterior mandible	N/A Clinical diagnosis (bone exposure post-extraction)	PBMT (660 & 808 nm, 100 mW, 10s/point, 1 J/point) + aPDT (MB 0.01%, 660 nm, 50s, 5 J/point); weekly × 30 days; adjunct: clindamycin	None	Healing, recurrence, mucosal coverage, prosthetic rehab, QoL	1 year	Complete healing in 30 days, no recurrence, successful prosthetic rehab, improved function & QoL	
Campos et al. [31]	Case report	Brazil	1	57 y.o. female, post-RT SCC, mandibular ORN with fistula	Posterior mandible	N/A Clinical diagnosis (ORN = exposed devitalized bone >3 months)	PBMT (808 nm, 70 mW, 1 J/point) + aPDT (MB 0.01%, 660 nm, 40 mW, 120s, 4.8 J/point); every 15 days × 6 wks	None	Healing of fistula, resolution of edema/erythema	6 months	Edema/erythema resolved after 7 days; fistula healed in 6 weeks	
Faustino et al. [16]	Case report	Brazil	1	53 y.o. male, polymorphous adenocarcinoma palate, post-RT (74 Gy)	Soft tissue necrosis buccal	N/A Clinical diagnosis (soft tissue necrosis, mucositis)	PBMT (DMC Therapy XT, 660 nm, 4 J/cm² over 10 points; 3 sessions)	None	Healing of mucositis/necrosis	1.5 years	Soft tissue healing, but follow-up incomplete; late RT complications observed (caries, trismus, ORN)	
de Freitas et al. [15]	Case report	Brazil	1	59 y.o. female, post-RT SCC (66.6 Gy), advanced ORN with cutaneous fistula	Anterior mandible	N/A Clinical diagnosis (advanced ORN)	PBMT (660 & 808 nm, 4 J/point), aPDT (MB 0.01%, 660 nm, weekly × 8 mo), Er:YAG laser (2940 nm, 3 W, single surgical), Diode surgical laser (808 nm, 1.5 W); adjunct: prosthetic rehab	None	Healing of fistula, mucosal repair, oral rehabilitation	1 year	Successful healing, implant-supported prostheses placed, QoL improved; safe and effective multimodal laser approach	

Clinical Outcomes 

The RCT by da Silva et al. [10] included 22 patients with lesions distributed across the mandible (13 cases), the maxilla (four cases), and both the mandible and the maxilla (five cases). Patients were staged using the Marx classification [5], with 68% in Stages I-II and 32% in Stages III-IV. The PBMT group achieved 94.7% mucosal healing within one month, compared with 0% in the sham group (p < 0.001), with notable pain reduction and lower analgesic use. No adverse events were reported.

The retrospective study by Ferigatto et al. [26] involved 51 patients. Lesions were primarily located in the posterior mandible (69.81%), followed by the anterior mandible (20.75%) and the posterior maxilla (10%). Staging was not reported; diagnosis was based on clinical assessment. The PBMT+aPDT group had a clinical success rate of 75% (median healing time of 10 months), compared with 18% in the standard care group (median healing time of 115 months). Oral hygiene was associated with improved outcomes (p < 0.0001). Adverse events were not reported.

The case series by Ribeiro et al. [14] included 20 cases classified using the He et al. [27] system. Lesions were found in the maxilla (six cases, 30%), with one anterior and five posterior, and in the mandible (14 cases, 70%), with seven anterior and seven posterior. Staging showed eight lesions in Stage I, seven in Stage II, and five in Stage III. Healing times were reported as Stage I: 17 weeks, Stage II: eight weeks, and Stage III: 19 weeks. Total mucosal coverage was achieved in 80% of cases. All eight Stage I cases fully healed, while six of the seven Stage II cases fully recovered. In Stage III, two of five cases healed completely. Pain relief was reported in 65%, with no recurrence during a two-year follow-up.

Among the case reports, Magalhães et al. [9] treated a Marx Stage III lesion in the anterior mandible using a combined aPDT and PBMT protocol. The patient achieved complete mucosal healing and showed no recurrence after one year.

Campos et al. [31] reported a clinically diagnosed posterior mandibular ORN with an associated fistula. Treatment with PBMT and aPDT led to the resolution of oedema and erythema within seven days and complete healing of the fistula by six weeks.

Tateno et al. [30] presented a case of posterior mandibular ORN following dental extraction in a high-risk irradiated patient. Diagnosis was clinical. A 30-day course of weekly PBMT and aPDT sessions resulted in full mucosal healing, with no recurrence during one year of follow-up. The patient also underwent successful prosthetic rehabilitation.

Pedroni et al. [29] described a posterior mandibular lesion in a patient with a history of laryngeal cancer, xerostomia, and comorbidities such as smoking and diabetes. PBMT and aPDT protocols were administered, targeting both the lesion and salivary function. Imaging demonstrated bone regeneration, and the patient experienced pain relief, improved xerostomia, and better oral function.

Porcaro et al. [28] treated a posterior maxillary lesion in a patient with a history of 90 Gy radiotherapy. The patient underwent a single Er:YAG laser session with adjunctive antibiotic therapy. Complete resolution was observed after 12 months, with no recurrence or infection.

de Freitas et al. [15] reported on a case of advanced anterior mandibular ORN complicated by a cutaneous fistula. A multimodal approach using PBMT, aPDT, and both Er: YAG and diode surgical lasers led to complete mucosal healing and successful placement of implant-supported prostheses within one year.

Faustino et al. [16] described a case of buccal soft tissue necrosis, diagnosed clinically, in a post-radiotherapy patient. The patient received three sessions of PBMT, resulting in mucosal healing. They discontinued treatment and were lost to follow-up. After 1.5 years, the patient returned, and although healing of the buccal mucosa was confirmed, several late complications of radiotherapy, including radiation caries, trismus, and ORN, were observed.

**Table 3:** Clinical outcomes across included studies PBMT: photobiomodulation therapy; aPDT: antimicrobial photodynamic therapy; ORN: osteoradionecrosis; QoL: quality of life; CBCT: cone beam CT; LLLT: low-level laser therapy; Er:YAG: erbium:yttrium-aluminium-garnet

Study	Healing time	Pain reduction	Mucosal coverage	Bone regeneration	Recurrence / progression	Adverse events
da Silva et al. [10]	Healing Time: PBMT significantly accelerated mucosal healing. By day 14, 94.7% of patients in the PBMT group achieved complete mucosal coverage compared to none in the sham-PBMT group	PBMT reduced postoperative pain significantly. At day 7, only 21.1% of patients in the PBMT group reported pain compared to 66.7% in the sham-PBMT group. The need for analgesics was also lower in the PBMT group	Faster and more complete mucosal coverage was observed in the PBMT group. By day 14, nearly all patients in the PBMT group had complete epithelialization of the surgical site.	Bone exposure was evaluated at D7, D14, D21, and D28 Healing described primarily relied on clinical examination, measurement of bone exposure using a periodontal probe	No recurrence during follow-up	None reported
Ferigatto et al. [26]	Protocol 1: Median epithelialization time was 115 months. Protocol 2 (aPDT): Median epithelialization time was significantly shorter at 10 months for lesions treated exclusively with this protocol and 30 months for lesions treated with both protocols	Clinical success was defined as the absence of painful symptoms, which was achieved in 75% of lesions treated with protocol 2.	Protocol 2 demonstrated effectiveness in achieving mucosal coverage through epithelialization in 75% of lesions.	Mechanisms supportive of bone healing discussed; no direct assessment	Of the 9 lesions that did not epithelialize with protocol 2, 5 experienced ORN progression, including pathological fractures or orocutaneous communication	None reported
Ribeiro et al. [14]	Stage I lesions: Average of 17 weeks (34.38 sessions). Stage II lesions: Average of 8 weeks (16.17 sessions). Stage III lesions: Average of 19 weeks (39 sessions).	Pain was absent in 65% of cases after treatment. Pain reduction was observed immediately after initial sessions.	80% of lesions achieved complete covering of the bone exposure by intact oral mucosa. 20% of lesions achieved partial mucosal coverage, primarily in Stage III cases.	Healing described; imaging not used	No recurrence after 2 years	None reported
Magalhães et al. [9]	Complete healing observed within 1 month	Pain relief reported	Reepithelialization confirmed at 1 month	Clinically inferred; imaging not reported	No recurrence at 12 months	None reported
Campos et al. [31]	Fistula fully healed within 6 weeks	Pain relief reported	Fistula closure and mucosal healing noted	Not reported	No recurrence reported	None reported
Tateno et al. [30]	Full mucosal healing achieved by day 30	Pain relief described during treatment	Confirmed by clinical observation	Bone exposure resolved clinically; no imaging	No recurrence over 12-month follow-up	None reported
Porcaro et al. [28]	Complete healing observed by 12 months	Pain resolved	Flap epithelialized by 15 days post-op	Clinical evidence of bone healing and osteoid formation; no imaging	No recurrence noted	None reported
Pedroni et al. [29]	Progressive improvement over 5 months; significant healing at 1 year	Pain relief contributed to QoL improvement	No bone exposure; oral mucosa hydrated	Confirmed by CBCT	No recurrence reported	None reported
de Freitas et al. [15]	Significant healing at 2 months; full recovery by 11 months (post-surgery)	Pain symptoms improved during therapy	Full mucosal healing achieved	Radiographic confirmation of bone regeneration	No recurrence at 1 year	None reported
Faustino et al. [16]	Partial soft tissue healing after 3 PBMT sessions; long-term follow-up showed late complications	Early symptom relief reported	Soft tissue healed at 1.5-year re-evaluation	Not reported	ORN and late RT complications observed	Radiation-related complications, not therapy-related

Quality of the Included Studies 

Each included study was assessed using the appropriate JBI checklist for retrospective cohort studies, case reports, and case series, as well as the RoB 2 tool for the RCT.

The RCT by da Silva et al. [10] demonstrated a low risk of bias in randomisation, intervention adherence, and outcome completeness, but raised some concerns in the measurement and reporting domains. Specifically, outcomes included subjective measures (pain scores), and follow-up was limited to 28 days. Overall, the study was rated as “some concerns,” with potential bias favouring the experimental PBMT group. The retrospective study by Ferigatto et al. [26] adequately described exposures and outcomes and had sufficient follow-up (10-115 months). However, it lacked strategies to address confounding factors, had incomplete follow-up due to the COVID-19 pandemic, and did not utilise imputation methods for missing data. 

The case series conducted by Ribeiro et al. [14] comprised 20 ORN cases, characterised by clear inclusion criteria, consistent diagnosis using Marx staging, and a two-year follow-up. Outcomes were clearly described, but only descriptive statistics were reported, and no comparator group was available. The study was deemed suitable for inclusion, although with moderate quality.

The included case reports were generally of moderate to high quality. All reports clearly described demographics, history, clinical presentation, diagnostic methods, interventions, and post-treatment outcomes. Adverse events, however, were inconsistently reported, with several studies [15,28,29] lacking documentation of harms. Despite this, all case reports provided clear lessons.

**Table 4:** Summary of quality assessment of the included studies The RCT showed a generally low risk of bias in randomisation and adherence but had some concerns in subjective outcome measurement and selective reporting. The retrospective cohort study had adequate follow-up but lacked control of confounding and strategies for missing data. The case series offered detailed outcomes over a two-year follow-up but lacked analytical comparison. All case reports clearly described demographics, interventions, and outcomes; however, adverse event reporting was inconsistent. The full detailed checklists for each study are presented in Appendices D-G.

Study	Study type	Quality appraisal tool	Overall appraisal	Key limitations
da Silva et al. [10]	RCT	RoB 2	Some concerns	Short follow-up (28 days), subjective outcomes (pain), small sample size
Ferigatto et al. [26]	Retrospective cohort	JBI Checklist (Cohort)	Include with caution	Incomplete follow-up, no adjustment for confounding, retrospective design
Ribeiro et al. [14]	Case series	JBI Checklist (Case Series)	Moderate quality	No comparator group, descriptive stats only
Porcaro et al. [28]	Case report	JBI Checklist (Case Report)	High quality	No adverse events reported
Magalhães et al. [9]	Case report	JBI Checklist (Case Report)	High quality	Harms not reported
Pedroni et al. [29]	Case report	JBI Checklist (Case Report)	High quality	Harms not reported
Tateno et al. [30]	Case report	JBI Checklist (Case Report)	High quality	No significant limitations reported
Campos et al. [31]	Case report	JBI Checklist (Case Report)	High quality	No adverse events reported
Faustino et al. [16]	Case report	JBI Checklist (Case Report)	Moderate quality	Long interval in follow-up, radiation complications later observed
de Freitas et al. [15]	Case report	JBI Checklist (Case Report)	High quality	Harms not reported

Discussion

All predefined clinical outcomes, healing time, pain reduction, mucosal coverage, bone regeneration, recurrence or progression, and adverse events were addressed across the included studies, though with varying depth and consistency. Healing time was reported in nine of ten studies [9,10,14,15,26,28-31], mucosal coverage in eight [9,10,14-16,26,30,31], and pain reduction in six [9,10,14,16,26,29]. Bone regeneration was specifically documented in two studies [15,29], while recurrence or progression of ORN was addressed in six [10,14,15,26,29,30]. Adverse events were rarely reported or explicitly discussed.

Although this review includes 10 studies, only two [10,26] employed comparator or control groups, enabling relative assessments of intervention effectiveness. The remaining eight studies, case series, or case reports provide clinical observations but lack controls, limiting their ability to establish causality or generalisability. Therefore, the evidence should be interpreted cautiously, particularly when comparing outcomes across different designs.

Across all study types, laser-based therapies, particularly PBMT and aPDT, demonstrated a consistent trend of promoting mucosal healing, reducing pain, and supporting functional recovery in patients with ORN. The RCT by da Silva et al. [10] reported a significant difference in healing rate (94.7% vs. 0%) within just one month, underscoring the short-term efficacy of diode laser PBMT in a controlled setting. However, this finding was limited by the small sample size and short follow-up. Pain reduction was also notable in this study: only 21.1% of the PBMT group reported pain at day seven, compared with 66.7% in the sham group, and analgesic use was significantly reduced. No adverse events were reported. Thus, while the clinical assessment method used in the study is effective for tracking mucosal healing and visible bone exposure, it may not fully capture the intricacies of bone regeneration.

The retrospective cohort study by Ferigatto et al. [26] provided longer-term data, with a median follow-up of 115 months. The improved healing in the aPDT + PBMT group (75% success) compared to standard care (18%) suggests laser-based adjuncts offer a clinical benefit. However, nine lesions failed to epithelialise, and five progressed to complications such as orocutaneous fistula or fracture. Still, no treatment-related adverse effects were reported. Pain control and mucosal healing were generally better in patients receiving protocol 2 (PBMT + aPDT).

The case series by Ribeiro et al. [14] included 20 cases staged using He et al. [27], with 30% in the maxilla and 70% in the mandible. Total mucosal coverage was achieved in 80% of cases. Stage-specific healing times were Stage I: 17 weeks, Stage II: 8 weeks, Stage III: 19 weeks. Stage I lesions showed 100% complete healing, Stage II had 85.7%, and Stage III showed 40%. This supports a stage-dependent therapeutic response, in which earlier-stage ORN may respond more predictably to laser-based therapy than advanced disease. Pain reduction was reported in all patients, with 65% experiencing complete relief. No recurrences were noted after two years. This demonstrates a stage-dependent therapeutic response, where earlier-stage ORN may respond more predictably to laser-based therapy than advanced disease.

These trends were further reflected across individual case reports. Magalhães et al. [9] achieved complete mucosal healing within one month with no recurrence after one year, while Tateno et al. [30] and Campos et al. [31] reported healing within 30-45 days, with fistula resolution and improved soft-tissue recovery. Pedroni et al. [29] and de Freitas et al. [15] confirmed bone regeneration via imaging following combined PBMT and aPDT, and both patients underwent successful prosthetic rehabilitation. Porcaro et al. [28] observed resolution of ORN and sinusitis by 12 months following Er: YAG laser and flap reconstruction, with no recurrence. Pain relief was frequently noted [9,29-31], and adverse events were rare or absent. However, Faustino et al. [16] illustrate the risks of incomplete treatment and lack of follow-up: although soft tissue healed after three PBMT sessions, the patient later presented with ORN, trismus, and radiation caries. These cases, despite their heterogeneity, support the potential utility of laser-based therapies across a spectrum of lesion stages and anatomical sites.

Laser Intervention Protocols and Comparative Evidence

Laser types varied, with diode lasers at 660 and 808 nm most commonly used. PBMT parameters ranged from 0.5 to 4 J/point and power settings of ~100 mW, while aPDT was typically performed with 0.01% methylene blue activated at 660 nm [9,14,15,26]. Surgical lasers (e.g., Er:YAG, diode surgical) were used in more advanced or refractory cases [15,28].

Session frequency ranged from single applications to multiple weekly sessions over several months. However, heterogeneity in wavelength, energy fluence, irradiation time, and frequency persists. This lack of standardisation limits reproducibility and prevents dose-response modelling. Future protocols must include rigorous dosimetric documentation.

Only a few studies reported complete radiotherapy details (e.g., dose, fields, fractionation), despite their known impact on ORN risk and healing outcomes [15,16,28]. This missing data weakens inter-study comparisons, as irradiated tissues differ markedly in healing potential based on dose exposure (e.g., >70 Gy vs. <60 Gy). Table 5 summarises the laser protocols in the included studies.

**Table 5:** Laser intervention protocols for ORN management ORN: osteoradionecrosis, PBMT: photobiomodulation therapy, aPDT: antimicrobial photodynamic therapy, CHX: chlorhexidine, J/point: Joules per point, J/cm²: Joules per square centimetre, mW: milliwatts, s: seconds, W: watts, Er:YAG: erbium-doped yttrium-aluminium-garnet, CW: continuous wave

Study type	Study (year)	Laser type and wavelength	Settings	Combined therapy	Sessions	Comparator / control
Randomised controlled trial (n = 1)	da Silva et al. [10]	Diode (808 nm)	40 mW, 2.8 J/point, 100 J/cm²	PBMT only	4 weekly sessions	Sham PBMT (inactive 660 nm)
Retrospective study (n = 1)	Ferigatto et al. [26]	Diode (660 nm)	100 mW, 2 J/point	aPDT + debridement + CHX gel	Not standardized; up to 115 months	Standard care (curettage, irrigation, antibiotics)
Case series (n = 1)	Ribeiro et al. [14]	Diode (660 & 808 nm)	100 mW, 10–40 s/point	PBMT + aPDT + CHX mouthwash	Not specified; observed over 2 years	None
Case reports (n = 8)	Porcaro et al. [28]	Er:YAG (2940 nm)	5 W, 250 mJ, 20 Hz	Surgical only + antibiotics	Single surgical session	None
	Magalhães et al. [9]	Diode (660 nm)	100 mW, 90 s/point (aPDT); 10 s/point (PBMT)	PBMT + aPDT	Weekly after surgery	None
	de Freitas et al. [15]	Diode (660 & 808 nm), Er:YAG	PBMT: 4 J/point; Er:YAG: 3 W; Diode surgical: 808 nm, 1.5 W	PBMT + aPDT + surgical + prosthetic rehab	Weekly aPDT × 8 mo; single surgical	None
	Tateno et al. [30]	Diode (660 & 808 nm)	PBMT: 100 mW, 10 s/point; aPDT: 50 s, 5 J/point	PBMT + aPDT + clindamycin	Weekly × 30 days	None
	Campos et al. [31]	Diode (808 & 660 nm)	PBMT: 70 mW, 1 J/point; aPDT: 40 mW, 120 s	PBMT + aPDT	Every 15 days × 6 weeks	None
	Faustino et al. [16]	Diode (660 nm)	4 J/cm² over 10 points	PBMT only	3 sessions	None
	Pedroni et al. [29]	Diode (660 & 808 nm)	PBMT: 100 mW, 10 s; aPDT: 20 s, 2 J/point	PBMT + aPDT	Frequent sessions	None

Beyond ORN-specific studies, other systematic reviews have demonstrated PBMT’s regenerative mechanisms. Santinoni et al. [32] reported enhanced bone regeneration and radiographic healing in maxillofacial defects, while Enwemeka et al. [33] reported significant improvements in soft-tissue healing and pain control in a meta-analysis of 34 human studies. Although these studies excluded irradiated tissues, they provide mechanistic validation for PBMT’s effects. Nevertheless, comparable outcomes have been noted in medication-related osteonecrosis of the jaw (MRONJ), where PBMT and aPDT reduced symptom burden [34,35], reinforcing the cross-condition applicability of laser-based modalities.

Limitations

This systematic review has several limitations, notably the inconsistency in ORN classification systems, which complicates comparing study outcomes. While some studies used established frameworks such as those of Marx [5] or He et al. [27], others relied solely on clinical assessment, thereby reducing staging precision and potentially misrepresenting treatment effectiveness. The adoption of the 2024 ISOO-MASCC-ASCO staging framework [18], which incorporates radiographic findings, soft-tissue status, and functional impairment, provides a more comprehensive and clinically meaningful tool for standardising future research.

The overall quality of evidence was low, with most included studies being case reports or small case series lacking control groups, blinding, or randomisation. This restricted the ability to perform a meta-analysis or draw definitive conclusions. Additionally, the included studies were highly heterogeneous in terms of laser parameters, treatment duration, and outcome measures, which hindered direct comparison and synthesis. Furthermore, there was limited reporting on radiotherapy details (e.g., dose, fields), which are essential prognostic variables that could not be adjusted for. Lastly, the review was limited to English-language studies, which may have introduced language bias and excluded relevant data from other regions.

Future Directions

To strengthen the evidence base, future research should prioritise the design and implementation of well-powered, multicentre RCTs using standardised laser parameters and clearly defined ORN staging systems. Consistent documentation of radiotherapy exposure, lesion site, and patient-specific risk factors will enhance comparability and allow for subgroup analyses. Longer-term follow-up is also needed to assess recurrence rates, the durability of healing, and functional rehabilitation, including quality-of-life outcomes. In addition, basic and translational studies should investigate laser-tissue interactions in irradiated bone and soft tissues to refine dose-response models and guide clinical applications. 

This systematic review aligns with the 2024 ISOO-MASCC-ASCO guidelines [18], which advocate the use of validated staging criteria and integrated multidisciplinary management. Although laser-based therapies show promise, the guidelines emphasise that current evidence remains insufficient to support routine use of PBMT or aPDT for ORN treatment. These findings underscore the need for further validation through robust clinical trials before formal recommendations can be made.

In parallel, the MASCC/ISOO guidelines on oral mucositis [36] provide strong support for the use of PBMT in the prevention and management of mucositis in patients undergoing cancer therapy. Although mucositis and ORN differ in pathogenesis, the biological mechanisms underpinning PBMT, such as anti-inflammatory effects, mitochondrial activation, and tissue regeneration, suggest potential crossover benefits. However, direct extrapolation from mucositis studies to ORN remains limited due to differences in tissue damage severity and vascular compromise. 

Despite the overall low methodological quality of the included studies, this systematic review synthesises valuable preliminary evidence that may inform clinical decision-making in settings where high-level data are unavailable-similar to how early-stage data guided the adoption of emerging interventions in other domains, such as the use of PBMT in oral mucositis. While not sufficient to establish definitive recommendations, these findings may support cautious, evidence-informed integration of laser therapies as adjunctive options in multidisciplinary ORN management.

## Conclusions

Laser-based therapies show promising potential in managing and preventing ORN in post-radiotherapy head and neck cancer patients. While findings are encouraging, the current evidence is predominantly derived from studies with lower methodological rigour; most included studies were classified as Level IV or V evidence, consisting of small, uncontrolled observational reports. Across various study designs, these interventions demonstrate favourable outcomes, including enhanced healing, reduced pain, improved mucosal repair, and a better quality of life. However, the heterogeneity in laser protocols and the absence of standardised ORN staging in nearly half of the studies limit the ability to compare outcomes and assess disease progression consistently. Further high-quality, controlled studies with standardised parameters and long-term follow-up are needed to confirm efficacy, optimise treatment protocols, and guide clinical practice.

## Appendices

Appendix A 

**Table 6:** PICO framework for the research question LLLT: low-level laser therapy, PBMT: photobiomodulation therapy, Er:YAG: erbium-doped yttrium-aluminum-garnet, aPDT: antimicrobial photodynamic therapy, HBO: hyperbaric oxygen

Category	
Population (P)	Patients with a history of head and neck radiotherapy at risk of or diagnosed with osteoradionecrosis (ORN)
Intervention (I)	Laser-based therapies (e.g., LLLT, PBMT, Er:YAG, diode lasers, CO₂ lasers, aPDT)
Comparison (C)	Standard care, placebo/sham treatments, non-laser-based modalities (e.g., antibiotics, HBO)
Outcome (O)	Healing time, pain reduction, mucosal/bone healing, recurrence prevention, adverse events, and overall symptom relief

Appendix B 

**Table 7:** Search string Filters: humans, English, publication dates from 2010 to 2025

Database / source	Search strategy (Boolean + MeSH/Keywords)	Notes
PubMed	("Osteoradionecrosis"[Mesh] OR osteoradionecrosis[tiab] OR ORN OR "radiation-induced osteonecrosis") AND ("Laser Therapy"[Mesh] OR "Low-Level Light Therapy"[Mesh] OR photobiomodulation OR PBMT OR "low-level laser therapy" OR LLLT OR "photodynamic therapy" OR aPDT OR "antimicrobial photodynamic therapy" OR "surgical laser" OR "laser surgery") AND (jaw OR mandible OR maxilla OR oral)	Used MeSH terms and keywords; includes jaw-specific terms
Web of Science	TS=(("osteoradionecrosis" OR "jaw necrosis" OR ORN) AND ("laser therapy" OR "low-level laser" OR photobiomodulation OR "photodynamic therapy" OR "surgical laser")) AND PY=(2010–2025)	TS = Topic search (title, abstract, keywords); filtered by year
Scopus	TITLE-ABS-KEY(("osteoradionecrosis" OR "jaw necrosis" OR ORN) AND ("laser therapy" OR "low-level laser" OR photobiomodulation OR "photodynamic therapy" OR "surgical laser")) AND PUBYEAR > 2009 AND PUBYEAR < 2026	Focused on title and abstract; filters applied for date range
Google Scholar	"osteoradionecrosis" AND ("photobiomodulation" OR "low-level laser" OR PBMT OR aPDT) AND "head and neck"Advanced filters: title includes "osteoradionecrosis" and "laser" or "photobiomodulation", English language only	Grey literature; filtered manually by title relevance and language
ICTRP (WHO)	("osteoradionecrosis" OR ORN) AND ("laser therapy" OR PBMT OR photobiomodulation OR aPDT)	Four registered trials identified; screened for eligibility
ClinicalTrials.gov	("osteoradionecrosis" OR ORN) AND ("laser therapy" OR PBMT OR photobiomodulation OR aPDT)	One relevant protocol found; no results reported (excluded)

Appendix C 

**Table 8:** Summary of reasons for studies exclusion during screening and full-text assessment

Reason for exclusion	Number of studies (n)
Wrong study design	55
Not focused on ORN (e.g., wrong outcome)	18
Non-English language	16
Unrelated intervention (e.g., wrong drug)	6
In vitro or animal study	8
Report not retrieved	2
Unpublished or protocol-only studies	4
Total	109

Appendix D 

**Table 9:** Risk of Bias 2 (RoB 2) assessment: randomized controlled trial (RCT) by da Silva et al. Citation: [10]

Domain	Judgement	Comments	Direction of bias
Domain 1: Randomization process	Low risk	Randomization described; sealed envelopes used; baseline groups balanced. Sa small sample size may limit robustness.	–
Domain 2: Deviations from intended interventions	Low risk	Double-blinded design with sham control; low risk of deviations; analysis according to randomization.	–
Domain 3: Missing outcome data	Low risk	All participants followed up for 28 days; no losses reported.	–
Domain 4: Measurement of the outcome	Some concerns	Outcomes included objective mucosal healing but also subjective pain scores, which may be influenced by patient perception despite blinding.	Favours experimental
Domain 5: Selection of the reported result	Some concerns	Pre-specified analysis plan reported, but the study focused only on short-term outcomes (28 days) and multiple outcomes were analyzed.	Favours experimental
Overall risk of bias	Some concerns	Well-designed pilot RCT, but limited by small sample size, short follow-up, and potential subjectivity in outcomes.	Favours experimental

Appendix E 

**Table 10:** Joanna Briggs Institute (JBI) checklist for cohort/analytical studies: Ferigatto et al. Citation: [26]

Question	Comments	Answer
1. Were the two groups similar and recruited from the same population?	Yes – all participants were post-radiotherapy ORN patients, divided by treatment protocol (aPDT vs standard care).	Yes
2. Were the exposures measured similarly to assign people to both groups?	Interventions (aPDT protocol vs conventional care) consistently described and applied.	Yes
3. Was the exposure measured in a valid and reliable way?	Detailed diode laser parameters and aPDT method provided.	Yes
4. Were confounding factors identified?	Oral hygiene identified as protective; other factors (e.g., comorbidities, RT dose) not systematically addressed.	No
5. Were strategies to deal with confounding stated?	No adjustment or stratification methods reported due to retrospective design.	No
6. Were participants free of the outcome at start of study?	Yes – all had ORN at baseline, but none had achieved healing prior to treatment.	Yes
7. Were outcomes measured in a valid and reliable way?	Healing/epithelialization outcomes reported, but reliance on retrospective records may reduce consistency.	Yes
8. Was the follow-up time sufficient for outcomes to occur?	Yes – median follow-up 10–115 months.	Yes
9. Was follow-up complete, and if not, were reasons explained?	Some patients lost during COVID-19 pandemic; acknowledged by authors.	No
10. Were strategies to address incomplete follow-up used?	No imputation or statistical correction for missing data.	No
11. Was an appropriate statistical analysis used?	Comparative analysis with significance testing (p ≤ 0.0001).	Yes
12. Overall appraisal	Provides long-term evidence, but limited by retrospective design and incomplete follow-up.	Include (with caution)

Appendix F 

**Table 11:** Joanna Briggs Institute (JBI) checklist for case series: Ribeiro et al. Citation: [14]

Criterion	Appraisal
Were clear inclusion criteria applied?	Yes – all ORN patients post-radiotherapy included
Was the condition measured consistently?	Yes – Marx staging system used
Were valid methods used for case identification?	Yes – clinical and radiographic diagnosis
Were cases included consecutively?	Yes – 20 patients reported in full
Was complete inclusion of cases achieved?	Yes – all included
Were outcomes measured clearly and objectively?	Yes – healing, pain, infection, fistula remission
Was the follow-up long enough for outcomes to occur?	Yes – 2 years
Was an appropriate statistical analysis used?	No – descriptive only, no comparator
Overall judgment	Provides descriptive evidence with moderate follow-up, but lacks a comparator and analytic rigour

Appendix G 

**Table 12:** Joanna Briggs Institute (JBI) checklist for case reports

Authors, year	Demographics	History	Clinical condition	Diagnostics	Intervention	Post-intervention condition	Adverse events	Takeaway lessons	Overall appraisal
Porcaro et al. [28]	Yes	Yes	Yes	Yes	Yes	Yes	No	Yes	Include
Magalhães et al. [9]	Yes	Yes	Yes	Yes	Yes	Yes	Yes	Yes	Include
Pedroni et al. [29]	Yes	Yes	Yes	Yes	Yes	Yes	No	Yes	Include
Tateno et al. [30]	Yes	Yes	Yes	Yes	Yes	Yes	Yes	Yes	Include
Campos et al. [31]	Yes	Yes	Yes	Yes	Yes	Yes	No	Yes	Include
Faustino et al. [16]	Yes	Yes	Yes	Yes	Yes	Yes	No	Yes	Include
de Freitas et al. [15]	Yes	Yes	Yes	Yes	Yes	Yes	No	Yes	Include
